# Morphoproteomics and biomedical analytics confirm the mTORC2/Akt pathway as a resistance signature and activated ERK and STAT3 as concomitant prosurvival/antiapoptotic pathways in metastatic renal cell carcinoma (RCC) progressing on rapalogs: Pathogenesis and therapeutic options

**DOI:** 10.18632/oncotarget.9508

**Published:** 2016-05-20

**Authors:** Robert E. Brown, Jamie Buryanek, Varaha S. Tammisetti, Mary F. McGuire, Keri Csencsits-Smith

**Affiliations:** ^1^ Department of Pathology and Laboratory Medicine, The University of Texas Health Science Center at Houston McGovern Medical School, TX 77030, Houston, USA; ^2^ Diagnostic and Interventional Imaging, The University of Texas Health Science Center at Houston McGovern Medical School, TX 77030, Houston, USA

**Keywords:** renal cell carcinoma, rapalog therapy, immunohistochemistry, MTORC2, resistance

## Abstract

**Background:**

It has been proposed that resistance to rapalog therapies in renal cell carcinoma (RCC) is due to adaptive switching from mammalian target of rapamycin complex 1 (mTORC1) to mTORC2.

**Objective:**

To combine phosphoprotein staining and applied biomedical analytics to investigate resistance signatures in patients with metastatic RCC progressing on rapalog therapies.

**Design:**

We applied morphoproteomic analysis to biopsy specimens from nine patients with metastatic RCC who continued to show clinical progression of their tumors while being treated with a rapalog.

**Results:**

In patients who were on temsirolimus or everolimus at the time of biopsy, a moderate to strong expression of phosphorylated (p)-mTOR (Ser 2448) in the nuclear compartment with concomitant expression of p-Akt (Ser 473) confirmed the mTORC2 pathway. Concomitant moderate to strong nuclear expression of p-ERK 1/2 (Thr202/Tyr204) and p-STAT3 (Tyr705) was confirmed. Histopathologic changes of hypoxic-type coagulative necrosis in 5 cases as well as identification of insulin-like growth factor-1 receptor (IGF-1R) expression and histone methyltransferase EZH2 in all tumors studied suggested that hypoxia also contributed to the resistance signature. Biomedical analytics provided insight into therapeutic options that could target such adaptive and pathogenetic mechanisms.

**Conclusions:**

Morphoproteomics and biomedical analytics confirm mTORC2/Akt as a resistance signature to rapalog therapy in metastatic RCC and demonstrate activation of the prosurvival ERK and STAT3 pathways and involvement of hypoxic pathways that contribute to pathogenesis of such adaptive resistance. These results highlight the need for a novel combinatorial therapeutic approach in metastatic RCC progressing on rapalogs.

## INTRODUCTION

In 2007 and 2009, the U.S. Food and Drug Administration granted approval for the use of temsirolimus and everolimus, respectively in advanced renal cell carcinoma (RCC). These agents are similar in action to sirolimus (rapamycin) and are regarded as rapalogs. The clinical success of temsirolimus and everolimus against advanced RCC has been limited by the development of progression in metastatic sites while the patients are on such therapies. The median progression free survival of patients treated with temsirolimus has been reported at 3.8 months [1, 2] and that for everolimus ranges from 4.9 months to 11.2 months [3, 1, 4]. The mechanism of such resistance with continued progression has been postulated to be due to adaptive switching from the relatively rapamycin-sensitive mTORC1 to the mTORC2/Akt pathway; and indeed, there are experimental studies to support, at least in part, this concept [5–10]. The purpose of this study is five-fold: 1) to provide evidence using morphoproteomics that patients whose metastatic RCC is progressing on temsirolimus or everolimus at the time of biopsy have mTORC2/Akt pathway expression; 2) to document the concomitant expression of constitutively activated prosurvival ERK and STAT3 pathways in such specimens; 3) to provide histopathologic and imaging data and morphoproteomic findings that hypoxia in the microenvironment and the insulin-like growth factor pathway may be contributing to the adaptive resistance; 4) to confirm and illustrate by biomedical analytics, the interconnection between the mTORC2/Akt, ERK and STAT3, and hypoxic pathways and their link to adaptive mechanisms posed by both the therapy and the microenvironment; and 5) using biomedical analytics, to propose a combinatorial therapeutic approach that targets such resistance mechanisms in metastatic RCC.

## RESULTS AND DISCUSSION

**Figure 1:**
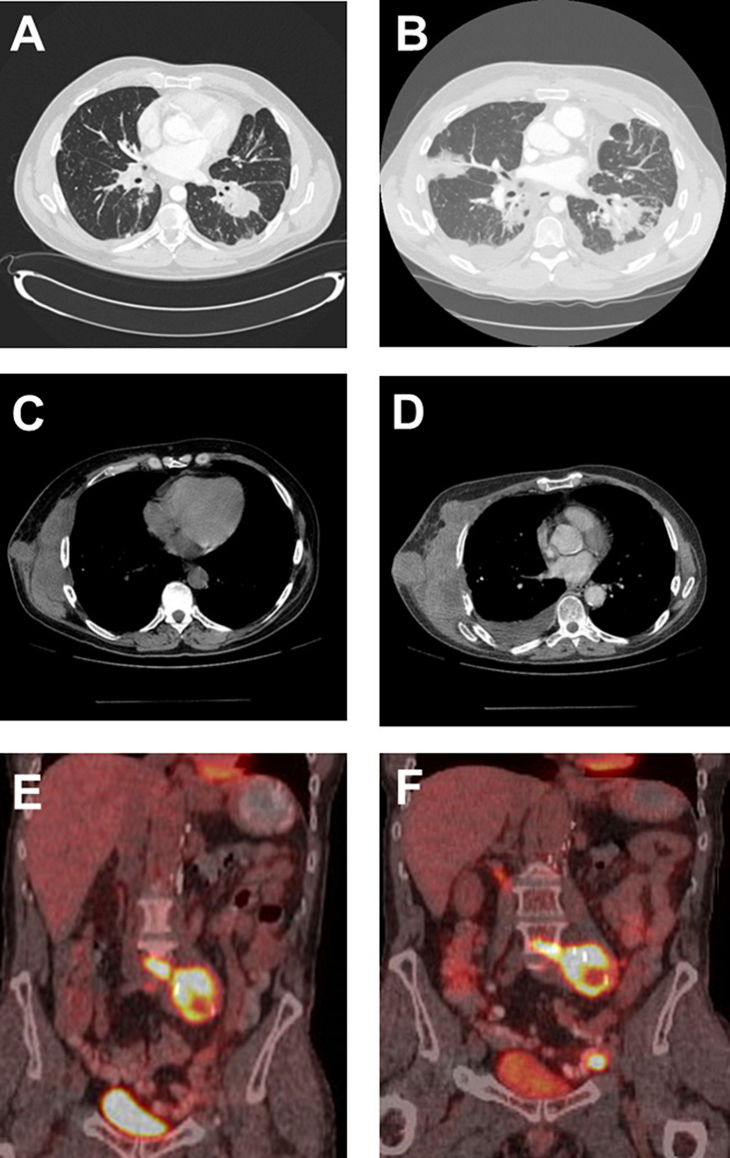
Radiographic imaging of baseline and progression of masses in RCC patients CT of the chest of patient 3 demonstrating lung nodules at baseline (**A**) and progression of the disease with new lesions, lymphangitic spread and worsened left pleural effusion (**B**). CT of the chest of patient 7 demonstrating right chest wall mass at baseline (**C**) and subsequent progression of disease with increase in size of this target lesion (**D**). A PET-CT fused coronal image of patient 4 showing intensely FDG-avid retroperitoneal lymph nodal mass (SUV of 12.1) at baseline (**E**) and subsequent development of new left external iliac lymph node (SUV of 22) as well as mild increase in the FDG activity of the retroperitoneal lymph nodal mass (SUV of 14.8) (**F**).

Clinical and demographic features of the patients in this study include the phenotype of their primary renal cell carcinoma, the metastatic site under treatment, and the type and duration of the rapalog therapy for the metastatic disease up to the time of progression and subsequent biopsy of the metastatic site, while on therapy (Table 1). Radiographic assessment, when applicable, confirmed the progressive nature of the metastatic disease in those patients on rapalog therapy and provided insight into the underlying conditions associated with such progression (Figure 1).

**Table 1:** Results of radiographic analyses to determine disease progression

ID	Exam	Response category	Criteria used	Findings
1	CT without IV contrast	Progressive Disease (PD)	RECIST 1.1	Progression based on non-target disease (lung nodules)
2	CT with IV contrast	Progressive Disease (PD)	RECIST 1.1	Progression based on new bony lytic lesions
3	CT with IV contrast	Progressive Disease (PD)	RECIST 1.1	Unequivocal progression based on numerous new lesions, new lymphangitic spread, worsened pleural effusion
4	PET	Progressive Disease (PD)	PERCIST	Progression based on development of new lesion
5	CT without IV contrast	Progressive Disease (PD)	RECIST 1.1	Progression based on development of new lesions and increase in size of several non-target lesions
6	CT with IV contrast and MRI with IV contrast for brain	Progressive Disease (PD)	RECIST 1.1	Progression based on development of new brain metastasis
7	CT without IV contrast	Progressive Disease (PD)	RECIST 1.1	Progression based on increase in size of target lesions
8	CT without IV contrast	Progressive Disease (PD)	RECIST 1.1	Progression based on new bony lytic lesion
9	CT without IV contrast	Progressive Disease (PD)	RECIST 1.1	Progression based on development of numerous new lesions

Abbreviations: RESIST = Response Evaluation Criteria in Solid Tumors.

PERCIST = PET Response Criteria in Solid Tumors.

**Figure 2:**
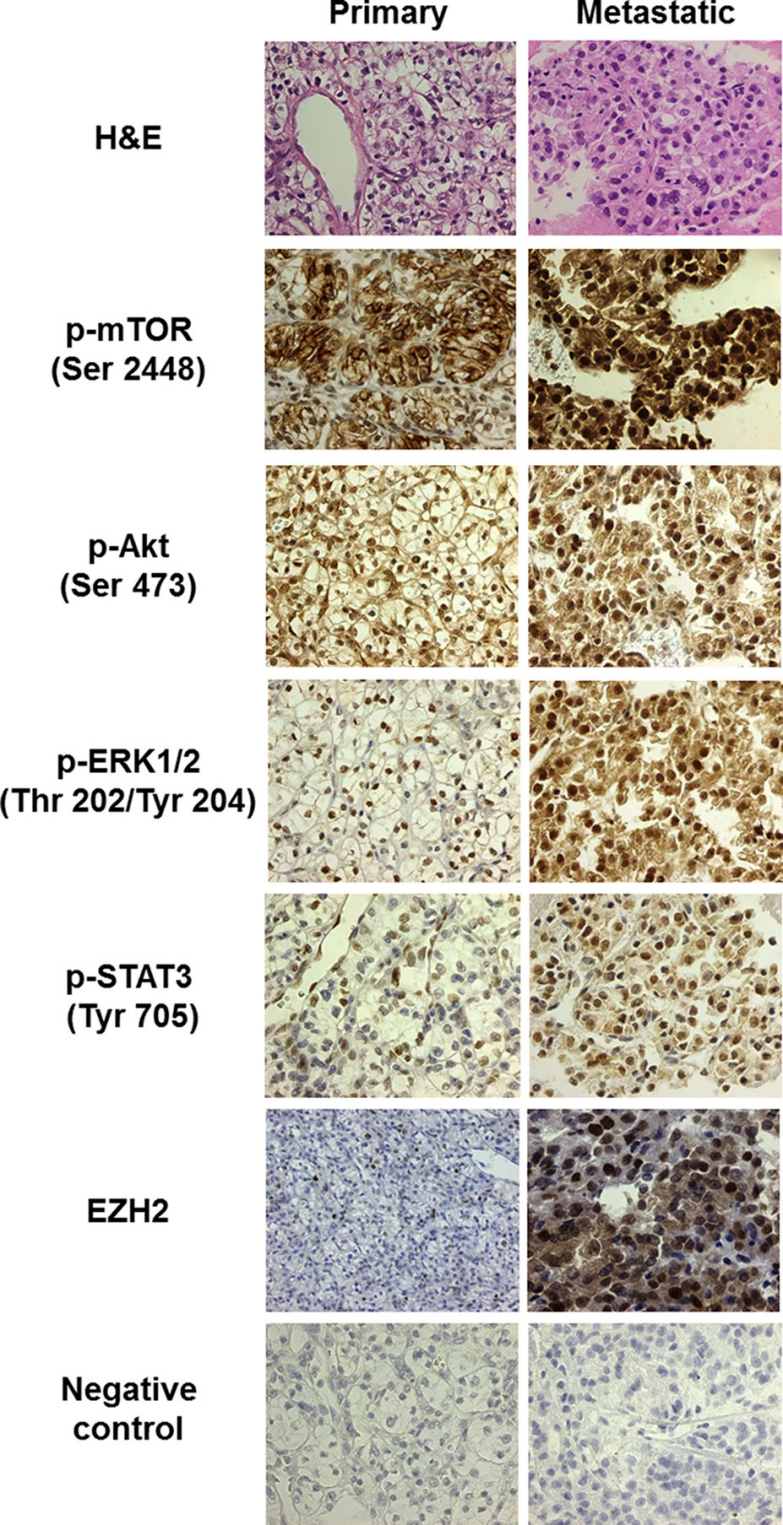
Morphoproteomic analysis reveals a shift to MTORC2 in metastatic tumor tissue Immunohistochemical probes specific for phosphorylated mTOR, p-Akt, p-ERK, and p-STAT3, as well as EZH2 were applied to formalin-fixed, paraffin-embedded sections of representative primary and metastatic tumor tissue biopsies from Patient 2. Immunohistochemical detection with the 3, 3′diaminobenzidine (DAB) chromogenic (brown) signal allowed for visualization of protein analytes in lesional and companionate cells. Note relative absence of nuclear expression of p-mTOR in the primary tumor, but nuclear translocation of this protein in the metastasis (indicative of MTORC2). All images are 400×, except for staining of EZH2 in the primary tumor, which is at 200× magnification to depict the relative paucity of positive nuclear staining.

Morphoproteomic analysis revealed that 6 of 6 patients on everolimus and 3 of 3 patients who were on temsirolimus (one of these patients also received everolimus) at the time of biopsy demonstrated moderate to strong up to 2 and 3+ signal intensity for p-mTOR (Ser 2448) and p-Akt (Ser473) in the nuclei of ≥ 50% of the tumor cells (Table 2, Figure 2).

**Table 2:** Morphoproteomic scoring of protein expression in biopsied tissue from patients with metastatic renal cell carcinoma progressing on rapalog therapy

ID	Classification	Rapalog	Months to biopsy	Biopsy site	Scores					
					p-ERK	p-Akt	p-mTOR	p-STAT3	IGF-1R	EZH2
1	Clear cell	Everolimus (+metformin)	26.5	Lung	Up to 3^+^	Up to 2^+^	Up to 3^+^	Up to 3^+^ (~100%)	± – 3^+^ (> 50%)	Up to > 50%
2	Clear cell	Everolimus (+metformin)	6.23	Bone	Up to 3^+^	Up to 2^+^	Up to 3^+^	3 (~50%)	1–2^+^ ~100%	10–20%
3	Conventional	Everolimus	0.30	Lung	Up to 2^+^	Up to 2^+^	Up to 2^+^	Up to 2^+^ (~30%)	2–3^+^ (> 50%)	Up to > 50%
4	Papillary	Everolimus	6.07	Lymph Node	Up to 3^+^	Up to 3	Up to 3^+^	Up to 3^+^ (~30%)	2–3^+^ (> 50%)	> 50%
5	Rare atypical	Everolimus	5.80	Lung	Up to 3^+^	Up to 3	Up to 3^+^	Up to 3^+^ (~100%)	2^+^ (~100%)	~20%
6	Clear cell	Everolimus (+metformin)	35.07	Bone	Up to 3^+^	Up to 3^+^	Up to 2^+^	Up to 3^+^ (~100%)	0–2^+^ (> 50%)	~20%
7	Sarcomatoid	Temsirolimus	2.73	Soft tissue	Up to 3^+^	Up to 3^+^	Up to 3^+^	3^+^ (> 50%)	1–3^+^	>50%
8	Clear cell	Everolimus, Temsirolimus	33.3	Soft tissue	Up to 3^+^	Up to 2^+^	Up to 2^+^	Up to 3^+^ (~50%)	0–1^+^ (< 50%)	< 10%
9	Clear cell	Temsirolimus	7.9	Lung	Up to 3^+^	1–2^+^	1–3^+^	1–3^+^ (~100%)	1–3^+^ (> 50%)	Up to > 50%

Additionally, moderate to strong nuclear expression of p-ERK 1/2 (Thr202/Tyr204) and p-STAT3 (Tyr705) were demonstrated in 9 out of 9 and 8 out of 9 cases, respectively (Table 2 and Figure 2). Notably, histopathologic examination of hematoxylin-eosin (H&E) stained sections as part of the morphoproteomic analysis revealed ischemic-type, coagulative necrosis in 5 out of 9 of the metastatic tumor biopsies, and total IGF-1R (Tyr1165/1166) was detected in all of the tumors up to 3+ signal intensity (Table 2). Expression of the histone methyltransferase EZH2 was also increased to at least 50% of tumor cells in 5 of the metastatic tumors (Figure 2, Table 2).

**Figure 3:**
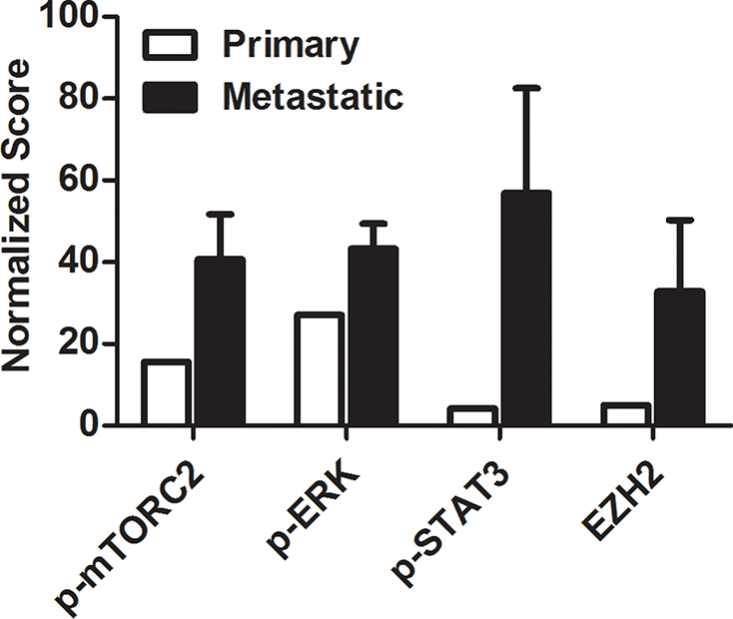
Normalized scoring of protein expression in metastatic tissue compared to primary tumor Morphoproteomic intensity scores were normalized and weighted using a customized algorithm that takes into account intensity of staining, overall cellular expression, and compartmentalization of the proteins. Scores for p-mTOR (Ser2448), p-Akt (Ser473), p-ERK1/2 (Thr202/Tyr204) and EZH2 staining in biopsied tissue were averaged for all nine patients (black bars) and compared to scores generated from the primary tumor of patient 2 (white bars).

In comparison to the morphoproteomic profile of a primary tumor the concomitant nuclear expression of p-mTOR (Ser2448) and p-Akt (Ser473) strongly indicates the presence of the relatively rapamycin-resistant mTORC2 pathway (Figure 3). To expand on this, mTOR phosphorylated on serine 2448 has been shown to bind to both raptor and rictor and, in the nuclear compartment, the complex is primarily with rictor, mTORC2 [11, 12]. Phosphorylation of Akt on serine 473 is attributed to mTORC2 [5, 7, 8]; and therefore, the expression of these two analytes in the nuclear compartment of the metastatic RCC is both concordant with and correlative of mTORC2 pathway signaling. Activation of the STAT3 pathway in all of the metastatic RCC biopsies in our current series accords with the earlier report by Horiguchi and colleagues of the high frequency p-STAT3 (Tyr705) nuclear expression in metastatic renal cell carcinoma and its association with progression and poor prognosis [13]. Potential activating factors shared by the mTORC2/Akt pathway and STAT3 pathway in this circumstance include hypoxia in the microenvironment and IGF-1R signaling in the tumor cells [14–17].

**Figure 4:**
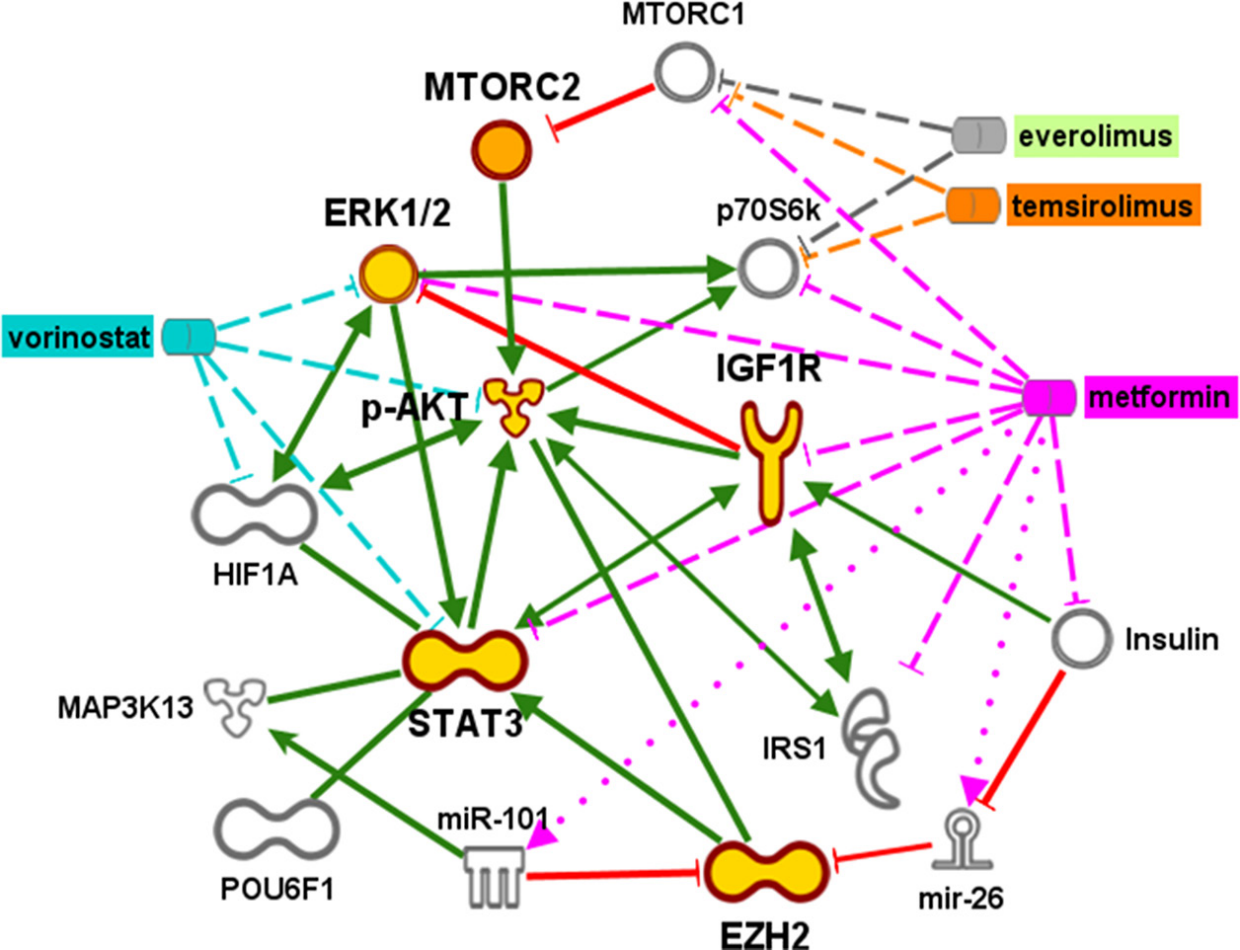
A graphical representation developed using biomedical analytics shows alternative survival pathways and potential therapies for metastatic tumors progressing on rapalog therapy Gold symbols indicate phosphoproteins whose weighted scores were input into Ingenuity Pathway Analysis to generate the most likely downstream interactions. Inhibition of MTORC1 activity via temsirolimus or everolimus interaction with the p70S6k subunit releases the brake on MTORC2. Subsequent signaling through pAkt and pERK is predicted to stimulate molecules associated with hypoxia (HIF1α, EZH2) and insulin growth receptors (IGFR, IGF1), via interaction through STAT3. Possible expression of transcription factor POUF6, as well as potential interactions with micro RNA (miR)101 and the micro RNA precursor mir-26 is associated with increased EZH2 expression. Green lines indicate binding or phosphorylation that leads to activation of the downstream protein and red lines indicate phospho-inhibition. Targets of proposed therapies using metformin (pink) and vorinostat (blue) are indicated by dashed or dotted lines. The dotted lines indicate activation, and dashed lines indicate inhibition.

Biomedical analytics applied to these morphoproteomic data provided confirmation of mTORC2 and the persistence of and collaboration with the prosurvival/antiapoptotic ERK and STAT3 pathways in all metastatic tumors clinically progressing on temsirolimus or everolimus therapy at the time of the biopsy (Figure 4).

While this evidence strongly suggests that the mTORC2/Akt pathway is characteristic of metastatic RCC, we cannot discount the possibility that this molecular signature is indicative of a primary tumor that metastasized. Indeed, expression of elevated staining for p-Akt (Ser473) in the primary tumor has been associated with metastatic disease in RCC [18]. On the other hand, Hager *et al*. reported that up to 30% of primary onset metastases with overexpression of total p-Akt (Ser 473) did not have concomitant over-expression in primary tumors [19]. The latter coincides with the previous report by one of us [REB] of p-Akt (Ser473) having the highest mean total expression score in metastatic RCC *vis-'a-vis* all phenotypes of primary RCC [20]. Moreover, O'Reilly and co-workers reported that everolimus (RAD001) therapy significantly increased the levels of p-Akt (Ser473) in skin or liver tumor biopsies from patients with colon or breast carcinoma [6] Hence, overexpression of p-Akt (Ser473) may be extremely variable in primary tumors and it is likely that this alone does not account for resistance to rapalog therapy.

Hypoxia in the microenvironment of the metastatic site of RCC is also likely to contribute to the activation of the mTORC2/Akt pathway. In support of a hypoxic influence is the report by Schultz and co-workers of both mTOR and hypoxia-induced pathways being activated in primary and metastatic clear cell RCC with higher levels of p-Akt (Ser473) in metastatic disease [21]. Relatedly, Hugonnet, et al. using 18F-fluoromisonidazaole PET/CT analysis found that patients with initially hypoxic metastases of RCC had a shorter time to disease progression (4.8 months vs. 11.3 months for other patients) [22]. Hypoxia is evident in some of our patients' metastatic tumors by virtue of the histologic findings of coagulative/ischemic type necrosis in 5 of the 9 biopsy specimens from metastatic sites.

In addition, the network derived from the molecular signatures of the metastatic tumors showed a possible convergence of prosurvival pathways associated with Akt/mTORC2 with hypoxic pathways indicated by increased IGFR1 expression (Figure 4). Indeed, the IGF pathway was expressed in our patients' metastatic tumors (Table 2). The increase in p-Akt (Ser473) expression via mTORC2 pathway signaling after rapamycin or rapalog administration has been linked to and requires the IGF-1R signaling pathway [5–7, 23].

Upregulation of the IGF-1R signaling pathway has been reported with von Hippel-Lindau (VHL) tumor suppressor loss in RCC and shown to culminate in enhanced Akt signaling and cellular invasiveness [24]. IGF-1R in RCC is associated with poor survival, particularly in patients with high expression levels [25–27], similar to the patients in this study.

Yet another factor in the development of rapalog resistance is the interaction of constitutively activated mTORC2/Akt with the STAT3 and ERK pathways (as illustrated in Figure 4) The prosurvival and antiapoptotic nature of constitutively activated mTORC2/Akt, STAT3 and ERK pathways is supported by the scientific literature and preclinical therapeutic strategies in renal cell carcinoma cells resulting in apoptosis when these pathways were inhibited [28–30]. An activated ERK pathway has been shown by Campbell, et al. “to be associated with advanced and aggressive pathologic features of renal tumors and predicts the onset of metastasis in patients with localized disease” [31]. Notably, Carracedo and colleagues [32] performed sequential biopsies from 10 cancer patients prior to and after the administration of RAD001 (everolimus) and 50% of these patients showed a marked increase in phosphorylation of ERK at threonine 202/tyrosine 204.

The ERK pathway can also be activated by hypoxia and the hypoxia-inducible factor (HIF) pathway [33, 34], the IGF-1R pathway [35] and via inhibition of the mTORC1 pathway by a rapalog [32]. A key link in this association might be the histone methyltransferase EZH2, the expression of which has been demonstrated to be enhanced by hypoxia through HIF1α-mediated transactivation and which may exert tumorigenic activity by inactivating tumor suppression genes [36]. We observed significant increase in EZH2 expression in metastatic tumors in our patients (Figures 2 and 3, Table 2). Moreover, biomedical analytics suggested that activation of STAT3 via EZH2 interaction can lead to activation of the transcription factor POU6F1, which has been associated with clear cell adenocarcinoma [37].

Recognition of the role of these prosurvival/antiapoptotic pathways and the mTORC2/Akt resistance to mTOR inhibitors in metastatic RCC has led to a focus on strategies that simultaneously target the Akt/mTOR, STAT3 and ERK pathways [38, 39]. Biomedical analytics graphically depicts the interaction of such agents against such pathways of resistance, survival and antiapoptosis in mRCC (Figure 4). Specifically, the HDAC inhibitor vorinostat inactivates the ERK and Akt pathways [40–44], and its clinical efficacy as a combination therapy in treatment of metastatic RCC is currently being evaluated [45].

Our analysis also demonstrates a use for metformin as a combination therapeutic. Metformin can target both the STAT3 and IGF-R signaling pathways to induce apoptosis [46–53]. Moreover, metformin can counteract the effects of hypoxia by upregulating miR-26a and miR-101, both of which repress EZH2 [54–57]. The potential benefit of metformin as a therapy was further highlighted by prolonged survival time observed in three of the patients analyzed in this study who received metformin together with everolimus (patients 1, 2, and 6, Table 2). The mean event-free survival time (EFT) was remarkably increased in these patients to 22.6 months (data not shown). This was in striking contrast to the EFT of 4.1 months observed in the 3 patients treated with everolimus alone (in agreement with previously reported EFS in patients with metastatic clear cell carcinoma [4]).

In summary, we have confirmed the constitutive activation of the mTORC2/Akt, STAT3 and ERK prosurvival/antiapoptotic pathways in metastatic RCC progressing on rapalog therapy. Moreover, we have provided insight into the microenvironmental and adaptive mechanisms leading to such resistance using clinical imaging, routine histopathology, morphoproteomics and biomedical analytics. Therapeutic options targeting such pathogenetic and adaptive mechanisms have been proposed for further consideration.

## MATERIALS AND METHODS

### Study population

With Institutional Review Board approval (HSC-MS-09-0549), we retrospectively reviewed the medical records on nine patients with metastatic renal cell carcinoma (RCC), all of whom were showing clinical progression on rapalog therapy at the time of the biopsy of the metastatic site and which occasioned the biopsy. All patients had nephrectomy. Six of the patients were receiving everolimus, two received temsirolimus only, and one (#8) was treated with both everolimus and temsirolimus (Table 1). The nature of the clinical progression was further characterized by imaging studies.

### Radiologic methods

Patients who underwent serial CT examinations were assessed for disease response or progression by RECIST 1.1 criteria (Response Evaluation Criteria in Solid tumors 1.1) [58] PERCIST criteria (PET response criteria in solid tumors) [59] was used to evaluate one patient who underwent PET examination only. MRI imaging of the brain was used when available. For each of the patients, the first available scan after the start of the targeted therapy was used as a baseline. The radiographic examinations that occurred immediately prior to the biopsy for morphoproteomics were evaluated and compared to the above mentioned first post-therapy baseline examinations for progression or response to therapy. Progressive disease was defined as at least 20% increase in the sum of the longest diameters of target lesions (taking as a reference the smallest sum on study and also the absolute increase in the sum by at least 5 mm) and/or development of new lesions. MASS (Morphology, Attenuation, Size and Structure) criteria were not used since not all patients received CT imaging with intravenous contrast due to poor renal function.

### Morphoproteomics

Morphoproteomic analysis [20, 60] was performed on all nine of the biopsy specimens. The histopathologic diagnosis on 8 tumors was conventional clear cell RCC and on 1, papillary RCC. Briefly, morphoproteomics involves the application of immunohistochemical probes to formalin-fixed, paraffin-embedded sections of representative tumor tissue. The analysis relies on bright-field microscopy and incorporates the following with respect to the protein analytes in lesional and companionate cells: their immunohistochemical detection with the 3, 3′ diaminobenzidine (DAB) chromogenic (brown) signal; quantification of their signal intensity on a scale of 0 to 3+; their microanatomical regions within the specimen and subcellular compartmentalization; and an assessment of their state of activation to include phosphorylation (p), compartmental translocation and functional grouping. Probes were applied for: mammalian target of rapamycin (mTOR), phosphorylated on serine 2448 (Cell Signaling Technology Inc., Danvers, MA); Akt, phosphorylated on serine 473 (Cell Signaling Technology Inc.); extracellular signal-regulated kinase (ERK 1/2), phosphorylated on threonine 202/tyrosine 204 (Cell Signaling Technology Inc.); signal transducer and activator of transcription (STAT3), phosphorylated on tyrosine 705 (Santa Cruz Biotechnology Inc., Santa Cruz, CA) insulin-like growth factor-1 receptor (IGF-1R [tyrosine 1165/1166], GenWay Biotech, Inc. San Diego, CA); and enhancer of zeste homolog-2 (EZH2, Cell Signaling Technology, Inc). The details of the morphoproteomic staining procedure have been previously described [20].

### Biomedical analytics

Biomedical analytics utilizes methods from computer science and mathematics to provide insight into the biology of processes [60, 61]. Its application in this study involved the integration of morphoproteomic analyses of the metastatic RCC with a focus on the resistance signature and adaptive prosurvival/antiapoptotic mechanisms relative to the tumor's progression while on rapalog therapy. Patient data were normalized and weighted by an algorithm customized for the morphoproteomic approach. The resultant scores for each of the protein analytes were entered along with their UNIPROT ID into Ingenuity Pathway Analysis (IPA, www.ingenuity.com). IPA was used to generate molecular signature data and pathways and these results were modified and augmented with info from National Library of Medicine's MEDLINE data base. Resistance and adaptive prosurvival/antiapoptotic signatures were confirmed and therapeutic options generated.
